# Risk Factors and Outcomes Associated with Gaps in Care in Children with Congenital Heart Disease

**DOI:** 10.1007/s00246-024-03414-y

**Published:** 2024-03-14

**Authors:** Michael B. Rosamilia, Jason Williams, Courtney A. Bair, Hillary Mulder, Karen E. Chiswell, Alfred A. D’Ottavio, Robert j. Hartman, Charlie J. Sang, Karl F. Welke, Michael J. Walsh, Timothy M. Hoffman, Andrew P. Landstrom, Jennifer S. Li, Lauren A. Sarno

**Affiliations:** 1 Duke University School of Medicine, Durham, NC USA; 2 Department of Pediatrics, Division of Pediatric Cardiology, Duke School of Medicine, Durham, NC USA; 3 Duke Clinical Research Institute, Duke University Medical Center, Durham, NC USA; 4 Department of Pediatrics, Division of Pediatric Cardiology, University of North Carolina at Chapel Hill Medical Center, Chapel Hill, NC USA; 5 Department of Pediatrics, Division of Pediatric Cardiology, East Carolina University, Greenville, NC USA; 6 Department of Surgery, Division of Pediatric Cardiothoracic Surgery, Atrium Health Levine Children’s Hospital, Charlotte, NC USA; 7 Department of Pediatrics, Division of Pediatric Cardiology, Wake Forest School of Medicine, Winston-Salem, NC USA; 8 Department of Cell Biology, Duke School of Medicine, Durham, NC USA; 9 Brody School of Medicine, East Carolina University, 115 Heart Drive, Greenville, NC 27834 USA

**Keywords:** Congenital heart disease, Health care utilization, Aftercare, Social determinants of health

## Abstract

Adults with congenital heart disease (CHD) benefit from cardiology follow-up at recommended intervals of ≤ 2 years. However, benefit for children is less clear given limited studies and unclear current guidelines. We hypothesize there are identifiable risks for gaps in cardiology follow-up in children with CHD and that gaps in follow-up are associated with differences in healthcare utilization. Our cohort included children < 10 years old with CHD and a healthcare encounter from 2008 to 2013 at one of four North Carolina (NC) hospitals. We assessed associations between cardiology follow-up and demographics, lesion severity, healthcare access, and educational isolation (EI). We compared healthcare utilization based on follow-up. Overall, 60.4% of 6,969 children received cardiology follow-up within 2 years of initial encounter, including 53.1%, 58.1%, and 79.0% of those with valve, shunt, and severe lesions, respectively. Factors associated with gaps in care included increased drive time to a cardiology clinic (Hazard Ratio (HR) 0.92/15-min increase), EI (HR 0.94/0.2-unit increase), lesion severity (HR 0.48 for shunt/valve vs severe), and older age (HR 0.95/month if < 1 year old and 0.94/year if > 1 year old; *p* < *0.05*). Children with a care gap subsequently had more emergency department (ED) visits (Rate Ratio (RR) 1.59) and fewer inpatient encounters and procedures (RR 0.51, 0.35; *p* < *0.05*). We found novel factors associated with gaps in care for cardiology follow-up in children with CHD and altered health care utilization with a gap. Our findings demonstrate a need to mitigate healthcare barriers and generate clear cardiology follow-up guidelines for children with CHD.

**Supplementary Information:**

The online version contains supplementary material available at 10.1007/s00246-024-03414-y.

## Introduction

Despite advances in surgical interventions and diagnostic capabilities over the past several decades, CHD remains a leading cause of death in children less than 10 years old [1, 2]. Even with surgery, many children with CHD may have residual disease and adverse sequela [3]. Thus, lifelong care by a congenital cardiologist is often required for patients with CHD. For adults living with CHD, the American College of Cardiology (ACC) and the American Heart Association (AHA) recommend follow-up surveillance with an adult CHD specialist at least every 2 years for most congenital lesions [4]. However, there are limited guidelines regarding follow-up in children. Some consensus groups have attempted to provide guidance on follow-up frequency in pediatric populations by specific cardiac lesion [5]. Despite follow-up recommendations, many CHD patients are not seen by pediatric cardiology specialists following hospital discharge, which can lead to increased morbidity and mortality [6–8].

CHD survival has been previously associated with several factors, including race, insurance status, and adherence to medical care [8–11]. Furthermore, there is evidence that an individual’s surroundings can have an impact on their health outcomes and likelihood of follow-up, as has been shown in asthma and preterm births [12–14]. Recently, several geospatially linked variables have been validated and have shown to strongly associate with healthcare access and outcomes [15]. Examples of validated, geospatially linked indices include Neighborhood Deprivation Index (NDI), Racial Isolation (RI), Education Isolation (EI), and drive time to the nearest cardiology clinic.

Despite the importance of aftercare for complex CHD, there are few studies with large samples showing factors associated with gaps in cardiology follow-up in children with CHD with subsequent differences in health outcomes. Using a large retrospective database spanning multiple tertiary care centers across the state of North Carolina (NC) and multivariable analysis techniques, we aim to determine socioeconomic, demographic, and medical factors associated with gaps in follow-up and the potential consequences of a gap in care, including increased morbidity, mortality, and health care utilization.

## Methods

### Data Access and Study Design

The North Carolina Congenital Heart Defect (NC-CHD) Surveillance Network was established in collaboration with the Centers for Disease Control and Prevention (CDC) in 2015 as part of a nationwide effort to characterize the epidemiology of CHD in the USA. Data were acquired via the NC-CHD Surveillance Network database, which includes data from 5 NC tertiary care hospital systems: University of North Carolina, Wake Forest Baptist Health, Atrium Health, Duke University Medical Center, and East Carolina University, as well as their satellite clinics, from 2008 to 2013. Data from Atrium Health, including demographic data, were excluded from this study due to lack of information on encounter type. Specifically, for this site, we could not distinguish outpatient visits with a cardiologist from other outpatient visits. For all other sites, demographic data were included for patients who had a cardiology encounter with an included diagnosis. Cases were identified using International Classification of Diseases (ICD), Ninth Revisions codes and were classified by hierarchical native anatomic complexity groupings similar to previously published algorithms based on the individuals’ hemodynamic severity and basic anatomy: severe disease, shunt disease, and valve disease [16]. Cases with an isolated code of 745.5 were excluded from this analysis since secundum atrial septal defect, a CHD, cannot be distinguished by ICD codes from patent foramen ovale (PFO), a normal variant and, therefore, inclusion of 745.5 may overestimate cases with CHD. Severe CHD included endocardial cushion defects, interrupted aortic arch, tetralogy of Fallot, total anomalous pulmonary venous return, transposition complexes, truncus arteriosus, and univentricular hearts. This study was approved by the Duke University Health System Internal Review Board and all the participating institutional review boards. All protected health information was maintained in a secure server and was deidentified for analysis. Details on data abstraction and processing are found in the **Supplemental Materials**. Support for the creation of the NC-CHD Surveillance Network database and statistical analysis was provided by the CDC grant 5 NU50DD004933-03–00.

### Patient Eligibility

Patients in the NC-CHD Surveillance Network database were included in this analysis if they were < 10 years old at the time of initial encounter and had a cardiology encounter with a diagnosis of CHD between January 1, 2008 and June 30, 2012. Similarly, patients who died within 18 months of their initial encounter were excluded due to insufficient time to assess follow up. A detailed description of the study cohort is displayed in Table 1. Additionally, a flow diagram of all excluded cases for each analysis can be found in Supplemental Fig. 1.

**Table 1:** Demographic cohort characteristics broken down by age range, including individuals < 1 year old and individuals 1- < 10 years old

Characteristic	Age < 1 year (N = 3797)	Age 1- < 10 years (N = 3172)	All Patients (N = 6969)
Age At Index Encounter-years	0.1 (0.0, 0.2)	4.7 (2.6, 7.1)	0.6 (0.0, 4.2)
Female	1931 (50.9%)	1496 (47.2%)	3427 (49.2%)
Race			
Asian, Native Hawaiian or Other Pacific Islander	55/3797 (1.4%)	61/3172 (1.9%)	116/6969 (1.7%)
Hispanic	580/3797 (15.3%)	403/3172 (12.7%)	983/6969 (14.1%)
Non-Hispanic Black or African American	1057/3797 (27.8%)	669/3172 (21.1%)	1726/6969 (24.8%)
Non-Hispanic White	1804/3797 (47.5%)	1836/3172 (57.9%)	3640/6969 (52.2%)
Other/Unknown	301/3797 (7.9%)	203/3172 (6.4%)	504/6969 (7.2%)
Racial Isolation Index	0.22 (0.13, 0.35)	0.19 (0.10, 0.32)	0.21 (0.12, 0.34)
Educational Isolation Index	0.78 (0.64, 0.86)	0.78 (0.63, 0.86)	0.78 (0.64, 0.86)
Neighborhood Deprivation Index	0.08 (-1.03, 1.24)	-0.12 (-1.40, 0.93)	0.00 (-1.14, 1.08)
Drive Time (minutes)	16.62 (10.25, 28.64)	18.01 (11.15, 29.20)	17.43 (10.63, 28.85)
Insurance			
Medicaid	1245/1954 (63.7%)	979/1900 (51.5%)	2224/3854 (57.7%)
Medicare	1/1954 (0.1%)	1/1900 (0.1%)	2/3854 (0.1%)
Private	600/1954 (30.7%)	825/1900 (43.4%)	1425/3854 (37.0%)
Self-Pay	1/1954 (0.1%)	1/1900 (0.1%)	2/3854 (0.1%)
Other	122/1954 (6.2%)	103/1900 (5.4%)	225/3854 (5.8%)
Site of Index Encounter			
01	1806 (47.6%)	1246 (39.3%)	3052 (43.8%)
02	498 (13.1%)	448 (14.1%)	946 (13.6%)
03	595 (15.7%)	751 (23.7%)	1346 (19.3%)
04	898 (23.7%)	727 (22.9%)	1625 (23.3%)
CHD Classification			
Severe	664 (17.5%)	717 (22.6%)	1381 (19.8%)
Shunt	1797 (47.3%)	1158 (36.5%)	2955 (42.4%)
Valve	1336 (35.2%)	1297 (40.9%)	2633 (37.8%)
Days from Index Encounter to End of Study	1415 (966, 1748)	1652 (1365, 2001)	1553 (1125, 1916)
Death	15 (0.4%)	15 (0.5%)	30 (0.4%)

Covariate characteristics are also described with continuous variables expressed as median [25th, 75th percentile] and discrete variables expressed as percentages unless otherwise noted

**Fig. 1:**
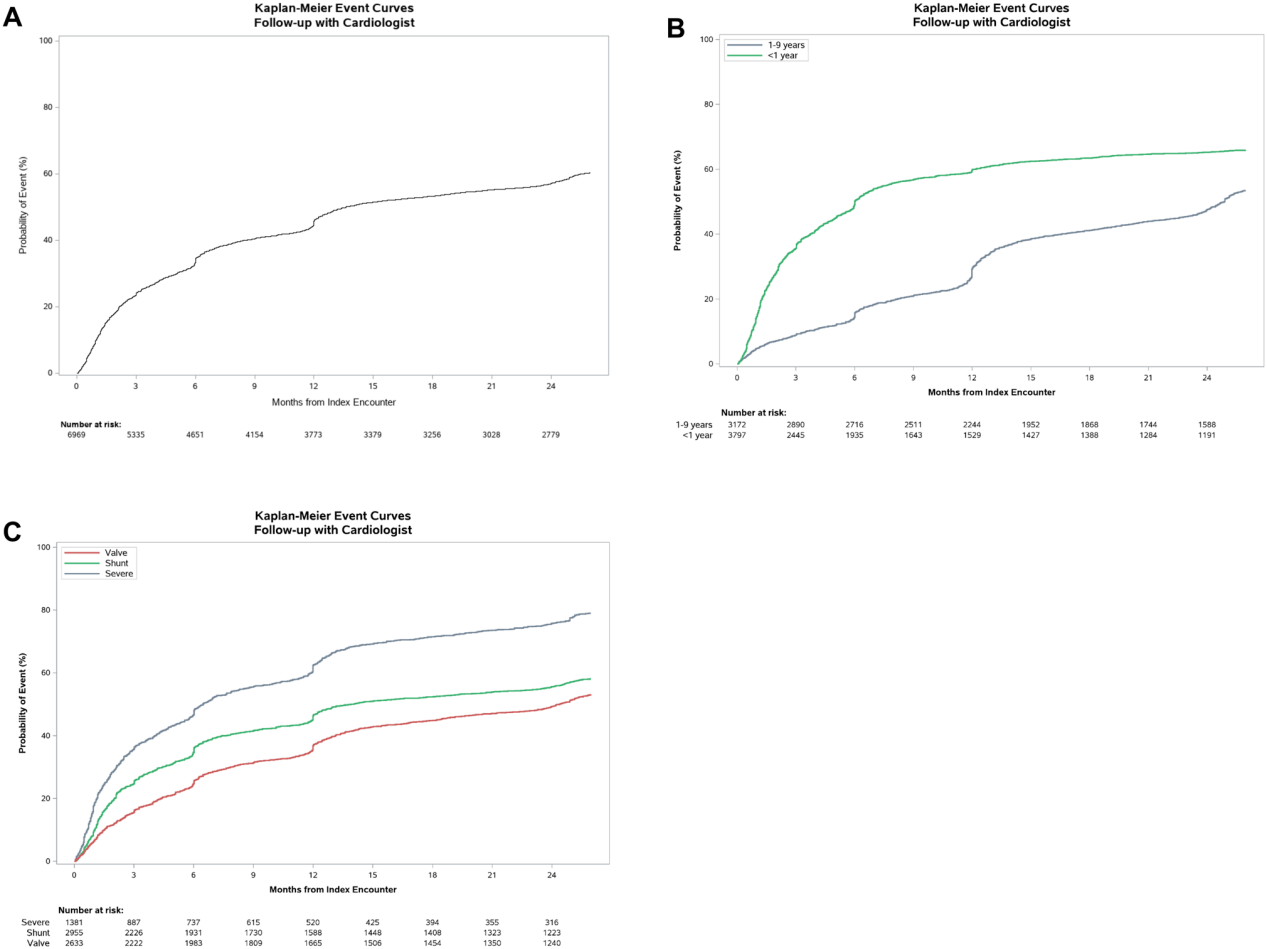
Kaplan–Meier curves depicting cardiology follow-up in children with CHD over the course of 2 years + 60 days from their index encounter overall in this cohort (**A**), by initial age at index encounter (**B**), and by category of CHD lesion (**C**). In panel B, age group refers to the age at the index encounter

### Outcomes

Based on ACC and AHA recommendations for cardiology follow-up in adults with CHD, a patient in our cohort was considered to have a gap in care if they did not have a second cardiology encounter, either inpatient or outpatient, within 2 years + 60 days of their initial encounter. We collected health care utilization encounters including the number of ED, inpatient, and outpatient visits and invasive and non-invasive procedures. Detailed information on categorization of invasive and non-invasive procedures can be found in the Supplemental Materials

### Statistical Analysis

Descriptive statistics on the cohort were calculated overall and by age category (< 1 year and 1–9 years). Continuous variables are presented as median (25th, 75th percentile) and categorical variables are count (percentage). Although the outcome of interest was cardiology follow-up within 2 years (+ 60 days) of index encounter, statistical analyses were conducted using time-to-event methods, to account for early censoring in patients without a full 2 years of data collection. Kaplan–Meier estimates for the percentage of patients who followed by within 2 years was calculated overall and by subgroups. Additionally, the cumulative percentage of patients with cardiology follow-up over time was visualized using Kaplan–Meier curves, and the log-rank test used to make comparisons between subgroups.

#### Analysis of Risk Factors for Gap in Follow-up

Measured covariates included age at index encounter, sex, and race/ethnicity as reported in the electronic medical record, EI, NDI, drive time to the nearest cardiology clinic, and CHD severity (non-severe (valve, shunt) or severe). NDI is a composite index that combines multiple socioeconomic variables, including occupation, education, and income [17]. EI quantitatively provides a measure of the ratio between non-college-educated individuals and college-educated individuals in a given residential area [18]. For each index, a higher number corresponds to worse deprivation or isolation. For example, a higher number in the NDI would indicate an area with a lower median income, a higher unemployment rate, fewer residents that are high school educated, and fewer households that are owner occupied [17]. Specific categorizations of CHD, by ICD code, can be found in the Supplemental Materials. Insurance status could not be included due to a high proportion of missing values (40%). Racial Isolation index (RI) (measuring the extent to which minority individuals can interact with majority individuals in a given residential area) was not included due to high collinearity with race/ethnicity. Certain variables, including EI, RI, and NDI are based on census tract. Address data were sourced from electronic medical records. The influence of all covariates on time to follow-up was analyzed using a multivariable, Cox proportional hazards model for time from index cardiology encounter to first subsequent cardiology encounter. Continuous variables were assessed for linearity using natural cubic splines and when found to be non-linearly related to follow-up, linear piece-wise splines were used in the model. The proportional hazards assumption was checked using weighted Schoenfeld residuals.

#### Analysis of Health Outcomes from Gap in Follow-up

A landmark analysis was used to evaluate differences in subsequent healthcare utilization between those who did and did not have a gap in cardiology follow-up during the first 2 years of follow-up. Patients were included in the utilization analysis if their follow-up extended at least 880-day post-index encounter. This period is longer than inclusion criteria for the other analyses to allow for ascertainment of cardiology follow-up status and additional time during which patients could have been seen in the health system. Counts of healthcare encounters for each patient began after the initial 2-year (+ 60 day) follow-up period. Utilization was evaluated using a negative binomial model with adjustment for age, sex, race/ethnicity, total drive time to nearest clinic, NDI, EI and CHD category, and log of post-landmark follow-up time as an offset.

Additionally, the incidence of death, severe cardiac dysfunction necessitating placement of a left ventricular assist device (LVAD), and cardiac transplantation occurrence during the post-landmark period were described, overall and by follow-up status. Patients were included in this descriptive analysis if they were alive and in the study at the 2-year (+ 60 day) mark.

Analysis code and selected deidentified datasets are available upon request. All analyses were performed by the Duke Clinical Research Institute (Durham, NC) using SAS version 9.4 (SAS Institute, Inc. Cary, NC).

## Results

### Study Population

In the study cohort, there were 6,969 unique children with CHD. This cohort included 3,797 (54.5%) children < 1 year old and 3,172 (45.5%) children 1- < 10 years old at their initial cardiology encounter. Approximately, half of the cohort was female (49.2%). A total of 24.8% of individuals were non-Hispanic Black and 14.1% were Hispanic. For all collected covariates, there was < 10% missing data except for insurance status, which was missing in > 40% of cases.

### Risks for Gap in Cardiology Follow-up

Overall, 60.4% of patients followed up with a cardiologist within 2 years + 60 days of their initial encounter. (Fig. 1A). Of children who were < 1 year old on initial encounter, 65.9% followed up compared with 53.5% of individuals 1- < 10 years old (p < 0.001) (Fig. 1B). The follow-up event rate by lesion severity was 79% for those with severe disease, 58.1% for those with a shunt lesion, and 53.1% with a valve lesion (p < 0.001) (Fig. 1C).

**Fig. 2:**
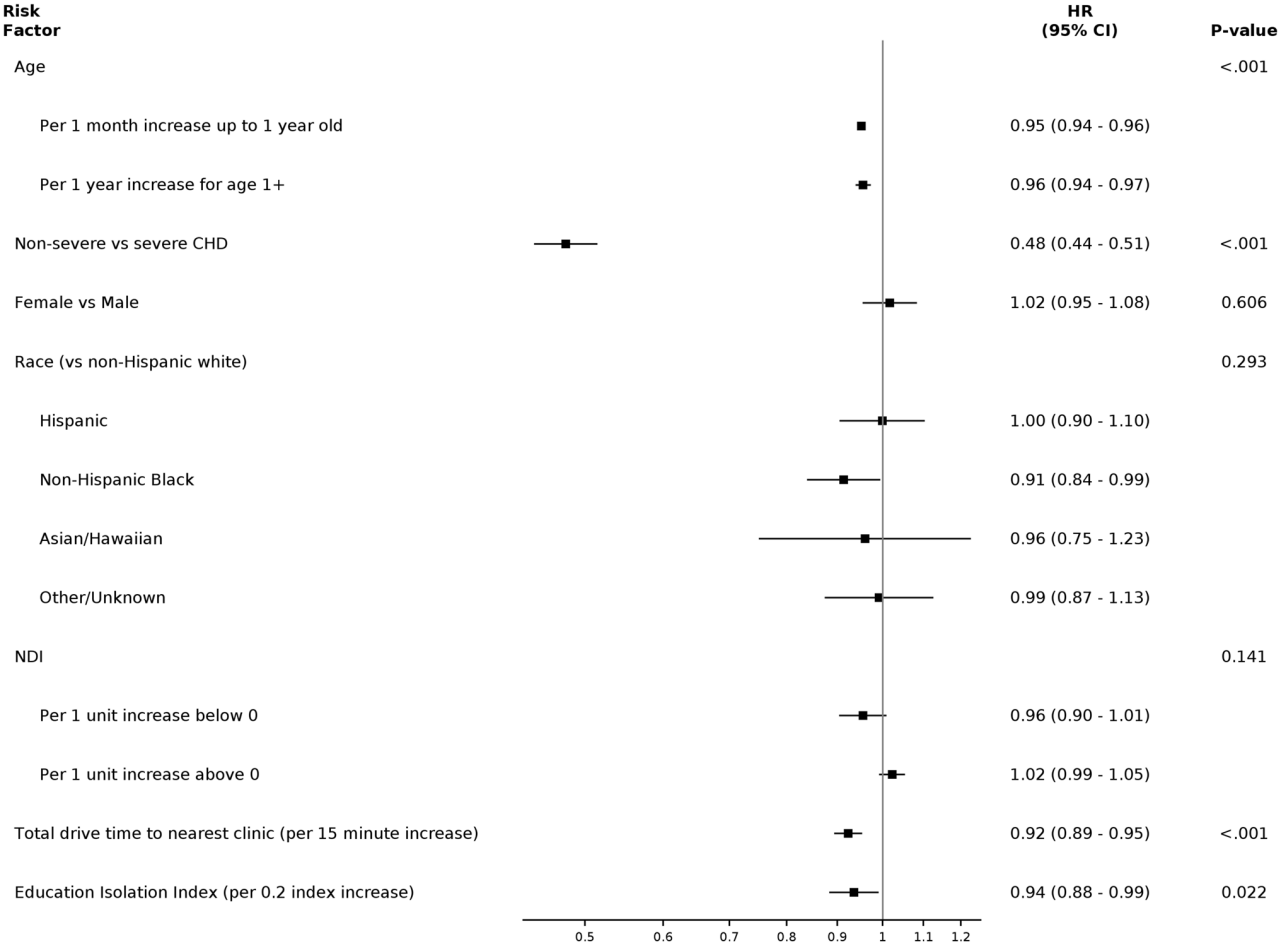
Forest plot showing adjusted hazard ratios for covariates associated with time to cardiology follow-up, with the 95% confidence interval. The hazard in this figure refers to the hazard of a child with CHD following up with a cardiologist subsequent to their index encounter. *EI—Educational Isolation; **NDI—Neighborhood Deprivation Index

To determine how patient demographics, disease severity, and socioeconomic factors were associated with time to cardiology follow-up, a Cox proportional hazards model was created, adjusting for multiple measured covariates. Factors associated with a significantly decreased likelihood of follow-up (*p* < *0.05*) included drive time to the nearest cardiology clinic, EI, age at initial encounter, and CHD classification of shunt or valve (non-severe) compared with a severe lesion. For drive time to the nearest cardiology center, every 15-min increase in drive time was associated with an 8% decrease in the rate of follow-up, meaning a lower probability of follow-up. Similarly, for every 0.2-unit increase in EI, there was a 6% decrease in the hazard of follow-up, with higher EI corresponding to worse isolation. For individuals < 1 year old, a 1-month increase in age at initial encounter corresponded to a 5% decrease in follow-up rate, whereas for individuals 1- < 10 years old, a 1-year increase in age at initial encounter was associated with a 4% decrease in follow-up hazard. Finally, individuals with non-severe CHD diagnoses had a 52% reduction in follow-up rate compared to those with a severe lesion. Factors not independently associated with a time to follow-up included sex, race/ethnicity, and NDI. All adjusted HRs are displayed in Fig. 2 and are shown in a tabular display in Supplemental Table 1.

### Health Care Utilization Differences After Gap in Cardiology Follow-up

**Fig. 3:**
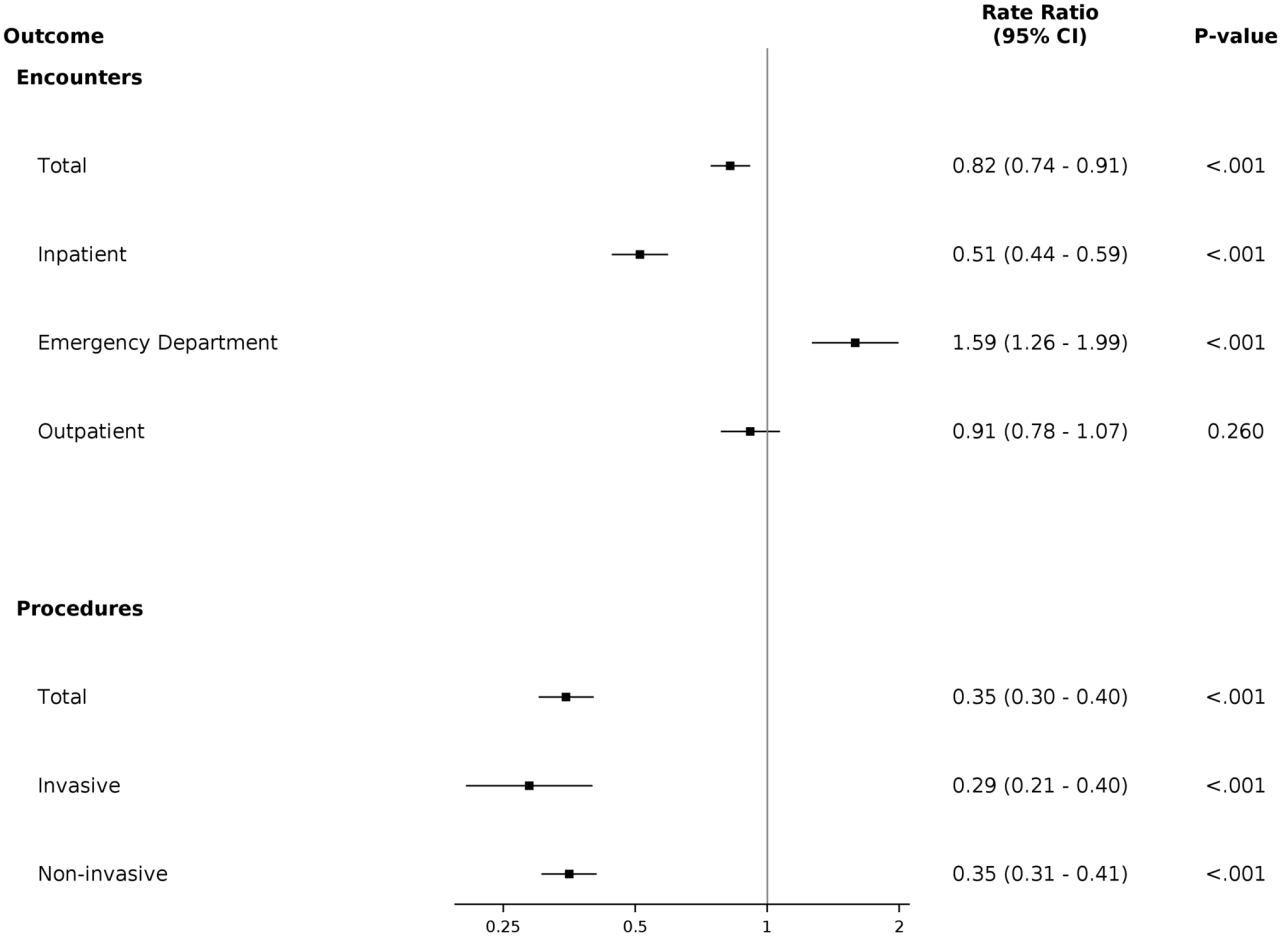
Forest plots depicting the adjusted RRs of utilization in individuals who do not follow-up versus individuals who do follow-up with a cardiologist within 2 years + 60 days of their index encounter for healthcare encounters (**A**) and procedures (**B**). *ED—Emergency Department

To determine effects of a gap in follow-up on subsequent interaction with the healthcare system, several utilization parameters were compared between children who did and did not receive cardiology aftercare within a 2-year + 60-day time frame from their initial encounter in a multivariable model. We found that patients who did not follow-up with a cardiologist within 2 years + 60 days of their initial visit were more likely to be seen in an ED (RR 1.59, *p* < 0.05) compared to patients who did follow-up. In contrast, this group was less likely to have an inpatient hospitalization (RR 0.82, *p* < 0.05) compared to those who followed up with no significant difference in outpatient encounter frequency (Fig. 3A).

As an additional utilization metric, the frequency of procedures was analyzed in people who did and did not follow-up in the 2-year + 60-day interval. In patients who did not have cardiology follow-up in that window, there was a significantly decreased likelihood of both invasive (RR 0.29) and non-invasive (RR 0.36) procedures (Fig. 3B). A tabular view of all adjusted RRs for utilization parameters can be found in Supplemental Table 2. Taken as a whole, we found differences in health care utilization for individuals who have gaps in cardiology follow-up in compared to those who did not, with a higher number of ED visits and a lower number of inpatient visits and procedures.

### Differences in Outcomes Based on Follow-up

To determine the incidence of severe events in children with CHD, three outcomes were examined: death, cardiac transplant, and placement of a LVAD. Overall, there was a low event rate of individuals experiencing any of the three outcomes after the landmark period (0.8%) There were no recorded cases of LVAD placement in this cohort. A higher proportion of individuals who did receive cardiology follow-up died (0.6%) or had a cardiac transplant (0.6%) compared to the group that did not follow-up with a cardiologist in that window (0.2% and 0.0%, respectively, Supplemental Fig. 2A) but this did not reach statistical significance. Additionally, a higher proportion of individuals with a severe CHD lesion experienced a poor outcome (2.3%) compared to those with a shunt (0.3%) or valve (0.4%) lesion (Supplemental Fig. 2B). Overall, severe outcomes were more common in individuals with cardiology follow-up with a low event rate for death, cardiac transplant, and LVAD placement.

## Discussion

In this retrospective cohort study of children with CHD in NC, we examined risk factors for gaps in cardiology aftercare, and differences in healthcare utilization and outcomes when gap in follow-up occurs. Factors associated with reduced rates of follow-up in cardiology care included increased drive time to a cardiology clinic, worse EI, older age at initial cardiology encounter, and non-severe CHD lesion. Healthcare utilization differs in individuals who had gaps in cardiology follow-up, with more subsequent ED visits and fewer inpatient encounters and procedures. Finally, in this cohort, there was a low event rate for death, heart transplant, and LVAD placement.

Our findings for risk factors associated with a reduced rate of cardiology follow-up add to the scope of known risks in children with CHD. There is previous evidence that as children with complex congenital diseases, including CHD age, they are less likely to attend regularly scheduled follow-up encounters [19–21]. Our results show that with increased age in infants, even on the scale of months, there is a substantial drop-off in likelihood of cardiology follow-up. The likelihood of follow-up continues to decline at a slower rate as children age from 1 to less than 10 years old. Explanations for why older children may be less likely to experience regular follow-up for congenital diseases include greater travel and scheduling constraints, including time in school, compared with younger children [22]. An additional risk for decreased follow-up, especially when moving from infancy to childhood, is the potential loss of peripartum insurance benefits. For example, in NC, neonates with mothers on Medicaid are automatically eligible for Medicaid but may lose this coverage after their first birthday [23]. Also, our finding that individuals with severe disease have a higher probability of follow-up with cardiology is concordant with previous findings that individuals whose daily lives are more impacted by their disease are more likely to follow-up [7]. Additionally, as a child ages, longer time to follow-up with spaced out clinic visits may be expected based on the patient’s clinical course. We acknowledge that age may not necessarily be a risk factor for loss to follow-up. Because of this, analyses adjusted for age and CHD severity.

A novel finding from this study is the substantial impact of drive time to a cardiology clinic on likelihood of follow-up in children with CHD. It has been previously shown that outcomes for hypoplastic left heart syndrome (HLHS) are worse for individuals who live further from tertiary care centers [24]. Our results show that drive time to a cardiology clinic has a major impact on cardiology follow-up, which may explain those previously described differences in outcomes. As about 40% of the NC population live in rural areas, there is an especially significant challenge to provide accessible cardiology care to all. Another novel finding of this study is the relationship between EI and likelihood of follow-up. Parental health literacy and education are prognostic factors of health outcomes in children with complex congenital disease [25]. We provide evidence that one way in which EI may impact outcomes involves greater likelihood of reduced rates of cardiology follow-up. Surprisingly, we did not find a significant association between length of time to follow-up and either race or NDI.

We found differences in healthcare utilization among children who had gaps in cardiology follow-up. Specifically, we found that children with CHD who have a gap in cardiology follow-up are more likely to be seen in the ED and less likely to have inpatient encounters or receive interventions. There are several possible explanations for why ED utilization differs in patients who have gaps in care. The first is that children with CHD who do not follow-up with cardiology may receive less focused subspecialty treatment and health optimization. As a child with CHD ages, especially when their congenital lesion is severe, their cardiovascular physiology has been shown to change, requiring medication adjustments or further intervention even when prior medical and/or surgical interventions were initially effective [26–29]. Patients who do not follow-up with cardiology within a 2-year period may miss out on these critical adjustments. Furthermore, signs of worsening clinical course, which may be amenable to intervention, may be missed. Any decrement in treatment efficacy, medical, or surgical could increase the likelihood of a CHD patient visiting the ED. A second potential explanation for the observed difference in ED utilization with reduced rates in cardiology follow-up is that subpopulations of individuals have been shown to regularly seek care in the ED and use it in place of an outpatient or primary care provider [30, 31]. This utilization pattern is both more burdensome on the healthcare system and leads to worse health outcomes [32]. Regardless of the relative contribution of these two hypothetical explanations, the increase in ED utilization in children with CHD who have a reduced rates of cardiology follow-up provides strong evidence in support of standardized of follow-up guidelines and outreach for vulnerable populations. It additionally provides an opportunity to reengage patients with pediatric cardiology care. The observed decrease in inpatient encounters and interventions may be representative of multiple phenomena, including decreased routine procedures, such as screening echocardiograms, in children who have a gap in care. Additionally, people with greater burden of disease and require more procedures may be more likely to follow-up outpatient. We found a low rate of death, cardiac transplantation, and LVAD placement in our cohort, with no recorded LVADs and < 1% of individuals experiencing death or transplant. This rate is considerably lower than in past studies of individuals with CHD, likely owing to improvements in surgical and medical management and a relatively young patient cohort [9]. We likely did not have power to detect difference in death and transplantation due to the very low event rate.

Overall, we find that there are strong predictors of reduced rates of cardiology follow-up in children with CHD, including drive time to the nearest cardiology clinic and EI. Both factors can be further explored via qualitative follow-up studies to identify specific barriers to care and potential interventions, including broader distribution of transportation vouchers or telemedicine when appropriate. Furthermore, we provide evidence for the creation of standardized follow-up guidelines for children with CHD given the observed increase in ED utilization in children without follow-up within 2 years, compared to children with follow-up.

## Limitations

In the absence of clear national guidelines, it is difficult to know when patients with CHD should follow up. Individual practitioners may recommend various intervals for their patients and the recommendations are likely based on many individual patient factors. To account for variations in recommendations, we chose a long follow-up period of 2 years with a 60-day grace period. Our analysis focused on identifying risk factors with a patient having their first follow-up visit in a reasonable time frame. We acknowledge that some patient might later be lost to follow-up and we are not capturing those patients. We also included lesion severity in our model as this is likely one of the most important factors clinicians use to determine follow-up guidelines. Regardless of when patients were recommended to follow-up our data showed differences in health care utilization in patients who had longer intervals to follow up.

There is an intrinsic potential bias in this study since people who do not follow-up with cardiology may have systematically lower data quality (more missingness) when compared with those who do follow-up. This limitation is mitigated by the fact that we only included individuals who have some form of recorded interaction with the healthcare system over a defined period. Another limitation is that we did not have information if a patient moved during the study and followed up with cardiology care outside of NC. Furthermore, CHD patients followed outside of the included major CHD centers would not be captured; some patients who were first seen at an included center and then followed up elsewhere (e.g., after an out-of-state move) would be erroneously labeled as lost to follow-up. However, based on 2013 US Census Bureau data showing the rate of total migration out of NC to be about 2.5% per year, it is unlikely that this significantly affected our results. Furthermore, 95% of pediatric cardiologists, all but one in NC, are captured in our database and thus it is unlikely that many in-state patients are getting appropriate CHD care elsewhere. If patients moved out of state and were considered to have a gap in follow-up, they likely also had lower or zero health care utilization. This limits our ability to detect greater utilization in those with gaps in care.

Our inability to include insurance status as part of our analysis limited our understanding of how this variable affects follow-up time and overall health care utilization in our specific population. As previously noted, there was a high percentage of missing insurance data in our population (40%). In addition to lack of insurance information, there may have been other important variables that were not included. Many care decisions both from the patient and provider perspective are complex and may not be entirely understood by the variables included in this study.

Additionally, while we chose a landmark study design to more effectively assess utilization and outcomes after gap in follow-up, we are still not able to assess causation (i.e., we cannot say that utilization changes occurred because of gap in follow-up). This study was performed in major, tertiary care hospital systems. The patient population seen in these systems may not be entirely representative of the total population of individuals with CHD.

## Conclusion

In this study, we have identified risk factors for gaps in cardiology follow-up in children with CHD and consequences of having a gap in care. We find that there are substantial predictors of gaps in cardiology follow-up based on patient factors, socioeconomic indicators, and barriers to healthcare. Our findings also suggest that regular follow-up with an outpatient cardiologist is important for optimal healthcare utilization in children with CHD. The creation of clear, concordant follow-up guidelines based on CHD lesion for infants and children is therefore an essential step in CHD care in the pediatric population. Additionally, continued efforts to improve the accessibility of cardiology care, especially in rural populations are needed.

### Supplementary Information

Below is the link to the electronic supplementary material.

Supplementary file1 (DOCX 228 kb)
