# Supplementary material for: Risk Factors and Outcomes Associated with Gaps in Care in Children with Congenital Heart Disease
Source: Pediatr Cardiol. 2024 Mar 14;45(5):976–85. doi: 10.1007/s00246-024-03414-y (PMC11056317; doi:10.1007/s00246-024-03414-y)
Supplement: Supplementary file 1 — Supplementary file1 (DOCX 228 kb) [file 246_2024_3414_MOESM1_ESM.docx]

**Risk factors and outcomes associated with gaps in care in children with congenital heart disease**

Michael B. Rosamilia MHS, Jason Williams MD, Courtney A. Bair BA, Hillary Mulder MS, Karen Chiswell PhD, Alfred D’Ottavio II BSE, BEE, Robert J. Hartman MD, Charlie J. Sang, Jr. MD, Karl F. Welke MD, MS, Michael J. Walsh MD, Timothy M. Hoffman MD, Andrew P. Landstrom MD, PhD, Jennifer S. Li MD, Lauren A. Sarno MD

**Supplemental Materials**

***Detailed Methods***

**Data abstraction and processing**

Data, including demographic, diagnosis, procedure, encounter, and death data were abstracted from the North Carolina Congenital Heart Disease (NC-CHD) database. All identifiable data was maintained on a secure server.

**Inclusion and Exclusion Criteria**

Inclusion criteria: In the NCCHD database, diagnosed with CHD (severe, shunt, valve), encounter at one of 4 North Carolina centers in study period (2008-2013), age <10 at index encounter and NC resident

Exclusion criteria: Diagnosis of PDA or secundum ASD alone (ICD9 747.0) or (ICD9 745.5) or index encounter occurring within 18 months of end of study or death

**CHD categorizations by ICD-9 code**

Severe:

745.0 (truncus arteriosus), 745.1 (transposition of the great vessels), 745.10 (complete transposition of the great vessels), 745.11 (double outlet right ventricle), 745.12 (corrected transposition of the great vessels), 745.19 (other transposition of the great vessels), 745.2 (Tetralogy of Fallot), 745.3 (common ventricle, including HLHS), 745.6 (endocardial cushion defects), 745.60 (endocardial cushion defects, unspecified), 745.69 (other endocardial cushion defects), 746.1 (tricuspid atresia), 746.01 (pulmonary valve atresia), 746.7 (HLHS), 747.41 (TAPVR), 747.11 (interruption of aortic arch)

Shunt:

745.4 (VSD), 745.5 (ASD, ostium secundum type), 745.61 (ASD, ostium primum type), 745.8 (anomalies of bulbus cordis or cardiac septal closure), 745.9 (unspecified defect of septal closure), 747.0 (PDA), 747.42 (PAPVR)

Valve:

746.0 (congenital anomaly of pulmonary valve), 746.00 (congenital pulmonary valve anomaly unspecified), 746.02 (congenital pulmonary valve stenosis), 746.09 (other congenital pulmonary valve anomalies), 746.2 (Ebstein’s anomaly), 746.3 (congenital aortic valve stenosis), 746.4 (congenital aortic valve regurgitation), 746.5 (congenital mitral stenosis), 746.6 (congenital mitral regurgitation), 746.81 (subaortic stenosis), 746.83 (infundibular pulmonic stenosis), 747.1 (coarctation of the aorta), 747.10 (coarctation of the aorta, preductal or post ductal) , 747.22 (atresia and stenosis of the aorta), 747.3 (congenital pulmonary valve anomaly), 747.31 (pulmonary artery coarctation and interruption), 747.39 (other anomalies of the pulmonary artery and circulation)

**Procedure categorizations**

Invasive: Aorta, Cardiac Biopsy, Cardiac Excision or Resection, Cardiac Imaging - Invasive Cath, Cardioversion, Conduit or Baffle, ECMO, Electrophysiology Procedure, Heart Transplant, Loop, Pacemaker or Pacing, Percutaneous Coronary Invervention (PCI), Pericardium, Repair of Congenital Vascular, Repair of Intracardiac CHD, Repair of Peripheral Vascular, Resuscitation, Septal Cath Procedure, Shunt, Surgical Coronary Revascularization, Thrombolysis, Valve Procedure, Valve Replacement, Vascular – Intracranial, Vascular – Peripheral, Vascular Imaging – Invasive, Ventricular Assist Device

Non-invasive: Cardiac Imaging - Noninvasive CT or MRI, Cardiac Imaging - Noninvasive Echo, Cardiac Imaging – Nuclear, EKG, Stress Test, Tilt, Vascular Imaging – Noninvasive,

***Supplemental Tables***

**Supplemental Table 1**

| **Risk Factor** | | **Hazard Ratio (95% CI)** | | **P-value** | |
| --- | --- | --- | --- | --- | --- |
| Age | |  | | <.001 | |
| Per 1 *month* increase for infants (<1 year) | | 0.95 (0.94 - 0.96) | |  | |
| Per 1 *year* increase for kids 1+ | | 0.96 (0.94 - 0.97) | |  | |
| Non-severe vs severe CHD | | 0.48 (0.44 - 0.51) | | <.001 | |
| Female vs Male | | 1.02 (0.95 - 1.08) | | 0.606 | |
| Race (vs non-Hispanic white) | |  | | 0.293 | |
| Hispanic | | 1.00 (0.90 - 1.10) | |  | |
| Non-Hispanic Black | | 0.91 (0.84 - 0.99) | |  | |
| Asian/Hawaiian | | 0.96 (0.75 - 1.23) | |  | |
| Other/Unknown | | 0.99 (0.87 - 1.13) | |  | |
| NDI | |  | | 0.141 | |
| Per 1 unit increase below 0 | | 0.96 (0.90 - 1.01) | |  | |
| Per 1 unit increase above 0 | | 1.02 (0.99 - 1.05) | |  | |
| Total drive time to nearest clinic (per 15 minute increase) | | 0.92 (0.89 - 0.95) | | <.001 | |
| Education Isolation Index (per 0.2 index increase) | | 0.94 (0.88 - 0.99) | | 0.022 | |

**Supplemental Table 2**

|  | | | **Unadjusted** | | **Adjusted** | |
| --- | --- | --- | --- | --- | --- | --- |
| **Outcome** | **Gap in Follow-up @ 2 years Rate (Total Events)** | **Cardiologist Follow-up Visit(s) Rate (Total Events)** | **Rate Ratio (95% CI)** | **P-value** | **Rate Ratio (95% CI)** | **P-value** |
| Total Encounters | 14.8 (9,959) | 18.8 (18,225) | 0.73 (0.66 - 0.80) | <.001 | 0.82 (0.74 - 0.91) | <.001 |
| Inpatient Encounters | 2.8 (1,914) | 6.9 (6,712) | 0.43 (0.38 - 0.50) | <.001 | 0.51 (0.44 - 0.59) | <.001 |
| Emergency Department Encounters | 1.2 (805) | 0.9 (863) | 1.26 (1.01 - 1.58) | 0.039 | 1.59 (1.26 - 1.99) | <.001 |
| Outpatient Encounters | 10.8 (7,240) | 11.0 (10,650) | 0.85 (0.74 - 0.99) | 0.035 | 0.91 (0.78 - 1.07) | 0.260 |
|  |  |  |  |  |  |  |
| Total Procedures | 4.1 (2,723) | 10.3 (9,955) | 0.34 (0.30 - 0.40) | <.001 | 0.35 (0.30 - 0.40) | <.001 |
| Invasive Procedures | 0.5 (334) | 1.6 (1,592) | 0.28 (0.21 - 0.39) | <.001 | 0.29 (0.21 - 0.40) | <.001 |
| Non-invasive Procedures | 3.6 (2,389) | 8.7 (8,373) | 0.36 (0.31 - 0.41) | <.001 | 0.35 (0.31 - 0.41) | <.001 |

**Supplemental Table 3**

|  | | **Follow-up at 2 Years** | |
| --- | --- | --- | --- |
| **Outcome** | **Overall (N=6,246)** | **Gap in Follow-up (N=2,501)** | **Cardiologist Follow-up Visit(s) (N=3,745)** |
| **Overall** |  |  |  |
| Death, HTx or LVAD | 47 (0.8%) | 6 (0.2%) | 41 (1.1%) |
| Death | 26 (0.4%) | 5 (0.2%) | 21 (0.6%) |
| HTx | 23 (0.4%) | 1 (0.0%) | 22 (0.6%) |
| LVAD | 0 (0.0%) | 0 (0.0%) | 0 (0.0%) |
| **Disease Severity: Severe** |  |  |  |
| Death, HTx or LVAD | 29/1257 (2.3%) | 3/269 (1.1%) | 26/988 (2.6%) |
| Death | 17/1257 (1.4%) | 2/269 (0.7%) | 15/988 (1.5%) |
| HTx | 14/1257 (1.1%) | 1/269 (0.4%) | 13/988 (1.3%) |
| LVAD | 0/1257 (0.0%) | 0/269 (0.0%) | 0/988 (0.0%) |
| **Disease Severity: Shunt** |  |  |  |
| Death, HTx or LVAD | 9/2623 (0.3%) | 3/1115 (0.3%) | 6/1508 (0.4%) |
| Death | 6/2623 (0.2%) | 3/1115 (0.3%) | 3/1508 (0.2%) |
| HTx | 3/2623 (0.1%) | 0/1115 (0.0%) | 3/1508 (0.2%) |
| LVAD | 0/2623 (0.0%) | 0/1115 (0.0%) | 0/1508 (0.0%) |
| **Disease Severity: Valve** |  |  |  |
| Death, HTx or LVAD | 9/2366 (0.4%) | 0/1117 (0.0%) | 9/1249 (0.7%) |
| Death | 3/2366 (0.1%) | 0/1117 (0.0%) | 3/1249 (0.2%) |
| HTx | 6/2366 (0.3%) | 0/1117 (0.0%) | 6/1249 (0.5%) |
| LVAD | 0/2366 (0.0%) | 0/1117 (0.0%) | 0/1249 (0.0%) |

***Supplemental Figures***

14,847 children <10 with CHD

*[-3,156] Excluded due to <18 months of follow-up*

*[-3,557] Excluded due to residence outside of NC or index visit at CMC*

*[-1,165] Excluded due to “Other” CHD categorization*

6,969 children included for assessment of cardiologist follow-up

*[-723] Excluded due to <2 years + 60 days follow-up*

*[-1,024] Excluded due to <2 years + 60 days follow-up + 90-day utilization window*

6,246 children included for evaluation of subsequent health outcomes

5,945 children included for evaluation of subsequent healthcare utilization

**Supplemental Figure 1:** Flow chart depicting the number of individuals included and excluded after initial review of records, for risk factor modeling, and for utilization modeling


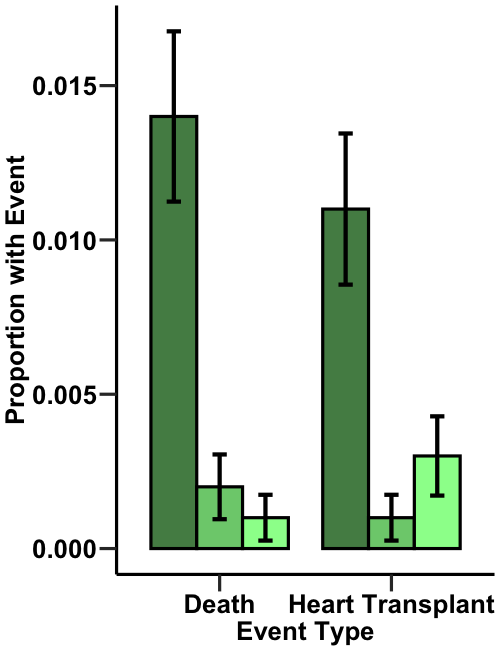

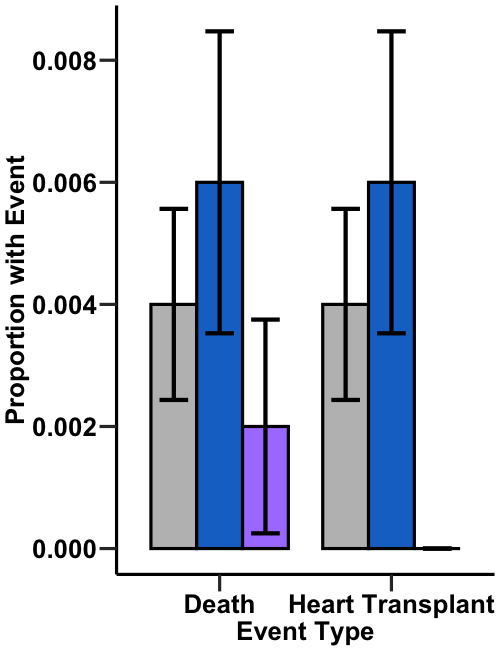


**Overall**

**With follow-up**

**Without follow-up**

**Severe**

**Shunt**

**Valve**

**A**

**B**

**Supplemental Figure 2:** Bar graphs depicting the proportion of individuals with an outcome of death or heart transplant after the 2 year + 60 day follow-up period by follow-up status (A) and CHD lesion type (B). Error bars represent a 95% confidence interval.
